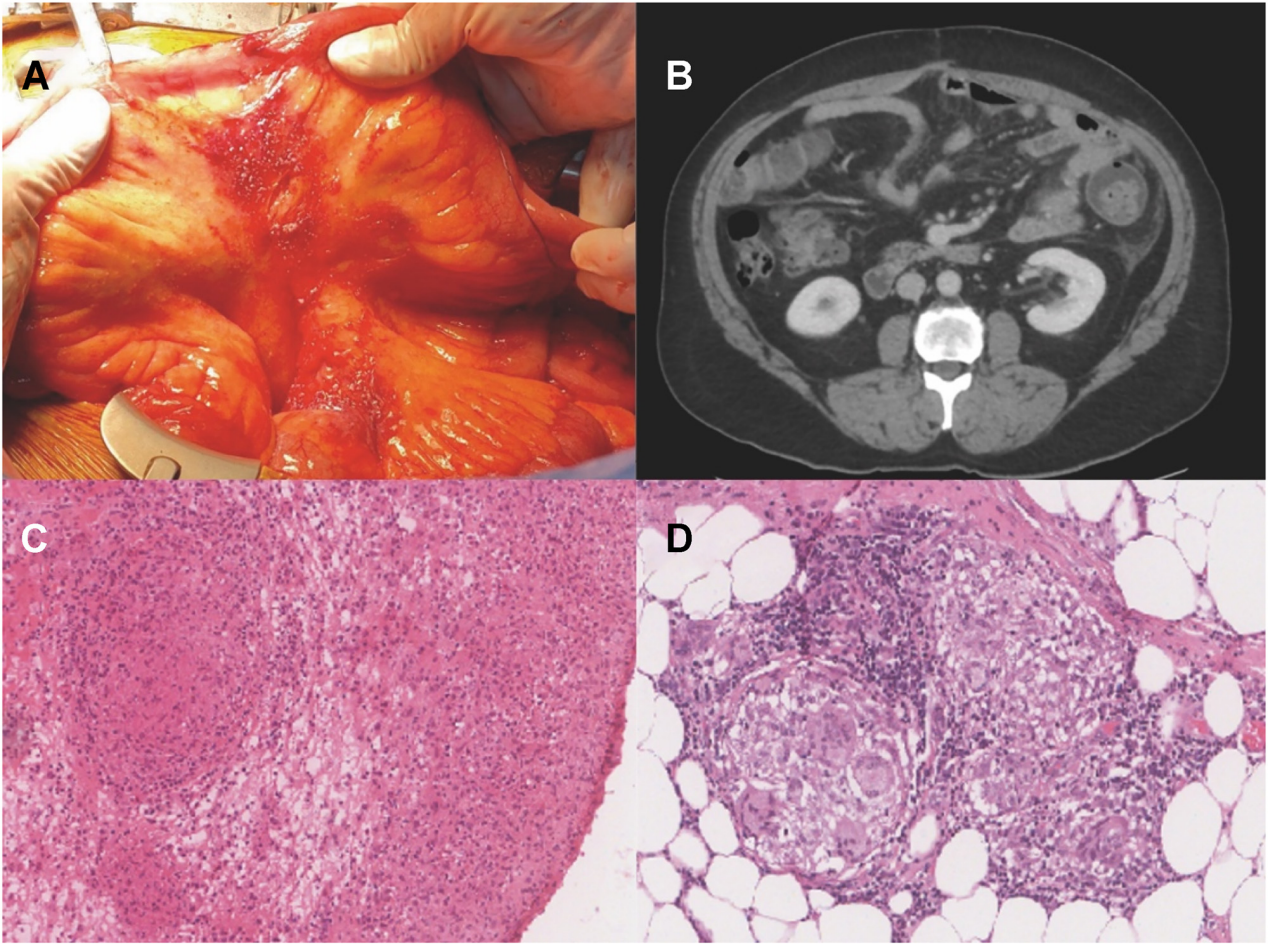# Abdominal tuberculosis mimicking peritoneal metastasis

**DOI:** 10.1515/pp-2023-0012

**Published:** 2023-06-15

**Authors:** Ragad I. Al Jazzar, Zeinah Sulaihim, Ahmad Alkhiary, Nayef Alzahrani

**Affiliations:** College of Medicine, King Saud bin Abdulaziz University for Health Sciences, Riyadh, Saudi Arabia; Department of Pathology, King Abdulaziz Medical City, Riyadh, Saudi Arabia; Department of Surgery, King Abdulaziz Medical City, Ministry of National Guard Health Affairs, Riyadh, Saudi Arabia

**Keywords:** extra-pulmonary tuberculosis, peritoneal metastasis, peritoneal tuberculosis

Peritoneal tuberculosis is a rare form of extrapulmonary tuberculosis. This challenge is attributed to its great mimicry of other peritoneal disease processes along with its nonspecific clinical presentation. We report a case of a 60-year old man, known case of rectosigmoid cancer with liver and lung nodules. Status post low anterior resection, sigmoidoscopy with primary anastomosis. The patient received multiple cycles of chemotherapy and radiotherapy. Patient underwent a cytoreductive surgery to eliminate the remaining peritoneal deposits. One year post initial cytoreduction, follow up scans revealed interval development of multiple peritoneal nodules with mesenteric congestion and irregularity highly suspicious of recurrent metastasis. Intra-operative findings were multiple macroscopic white/pink nodules. Histopathological biopsies were evaluated and revealed granulomatous inflammation, negative for malignancy. Resection of peritoneal nodules revealed fibrofatty tissue with chronic granulomatous inflammation mostly non necrotizing with a few necrotizing subtypes, negative for malignancy, negative Acid fast bacilli stain. The patient had a positive family history for TB, and was commenced on anti-TB regimen.

**Figure:**